# Combination Therapy of Extracorporeal Shock Wave Therapy and Platelet-Rich Plasma for Musculoskeletal Disorders: A Narrative Review

**DOI:** 10.7759/cureus.116717

**Published:** 2026-09-22

**Authors:** Toru Omodani

**Affiliations:** 1 Orthopaedics, Tokyo Advanced Orthopaedics, Tokyo, JPN

**Keywords:** combination therapy, eswt, extracorporeal shock wave therapy, musculoskeletal disorders, nonunion, platelet-rich plasma, prp, regenerative medicine, tendinopathy

## Abstract

Extracorporeal shock wave therapy (ESWT) and platelet-rich plasma (PRP) have each been used for musculoskeletal disorders, but the role of their combination remains incompletely defined.

This narrative review summarizes the PubMed-indexed evidence cited in the present manuscript regarding the biological rationale, current clinical evidence, treatment sequence, and remaining challenges of ESWT+PRP therapy. ESWT acts through mechanotransduction and may promote pain modulation, angiogenesis, tissue repair, and bone formation, whereas PRP provides growth factors that may support inflammation regulation, cell migration, angiogenesis, and extracellular matrix formation. Direct clinical combination studies are currently available mainly for patellar tendinopathy, lateral epicondylitis, partial rotator cuff tears, and aseptic nonunion of long bones. These studies suggest potential additive effects in early pain relief, functional recovery, and bone union.

In contrast, for Achilles tendinopathy and plantar fasciitis, the literature largely compares ESWT and PRP monotherapies, and direct evidence for the combination remains limited. Bone disorders show strong biological plausibility and provide the clearest direct clinical support for aseptic nonunion.

Overall, ESWT+PRP should currently be viewed as a promising but non-standardized strategy. Its clinical use appears most reasonable in selected chronic tendinopathies and biologically compromised bone healing, while standardized protocols and higher-quality comparative studies remain necessary.

## Introduction and background

Extracorporeal shock wave therapy (ESWT) is a non-invasive physical modality widely applied for musculoskeletal disorders, including tendinopathy, plantar fasciitis, calcific shoulder tendinopathy, and bone nonunions [1,2]. Its therapeutic foundation rests on mechanotransduction, a biological cascade through which acoustic pressure waves are converted into cellular biochemical signals [1,2]. The documented biological responses to ESWT include pain modulation with decreased substance P in the treated area, neovascularization, stimulation of cellular proliferation and protein biosynthesis, disruption of calcific deposits, and induction of osteogenesis [1,3]. Clinical devices generate either focused ESWT (fESWT), which achieves much higher intensity at a target deep within the tissue, or radial ESWT (rESWT, extracorporeal radial pressure wave therapy), a lower-intensity pressure wave generated by a pneumatic mechanism whose maximal intensity occurs at the applicator surface [1,3]. Because these modalities differ in their physical characteristics, they are treated as distinct wherever the source studies permit. Substantial clinical evidence supports ESWT monotherapy across several orthopedic indications, most notably calcific tendinopathy of the shoulder and delayed bone union or nonunion fractures, with generally favorable results also reported for chronic Achilles tendinopathy [1-3].

Concurrently, platelet-rich plasma (PRP) has emerged as a cornerstone of orthopedic regenerative medicine. Obtained through the centrifugation of autologous peripheral blood, PRP yields a supra-physiological concentration of platelets suspended in a small volume of plasma [4,5]. Upon activation, platelet alpha-granules release critical bioactive mediators, including platelet-derived growth factor (PDGF), transforming growth factor-beta (TGF-β), vascular endothelial growth factor (VEGF), fibroblast growth factor (FGF), and epidermal growth factor (EGF) [4,5]. These cytokines orchestrate local inflammatory regulation, cellular chemotaxis, angiogenesis, and extracellular matrix synthesis [4,5]. Nevertheless, PRP is not a monolithic product. Substantial disparities in baseline platelet enrichment factors, leukocyte preservation, red blood cell contamination, exogenous activation techniques, delivery volumes, injection numbers, and anatomical guidance methods across clinical studies necessitate meticulous appraisal of specific preparation characteristics [4,5]. The classification system proposed by Dohan Ehrenfest et al., categorizing autologous platelet concentrates into pure platelet-rich plasma (P-PRP), leukocyte- and platelet-rich plasma (L-PRP), pure platelet-rich fibrin (P-PRF), and leukocyte- and platelet-rich fibrin (L-PRF), remains vital for meaningful comparative analysis [6]. Systematic reviews have confirmed that while PRP monotherapy demonstrates favorable efficacy in chronic tendinopathies such as patellar tendinopathy and lateral epicondylitis, results remain inconsistent in Achilles tendinopathy and rotator cuff pathology [7-9].

Given that chronic recalcitrant tendinopathies and impaired fracture healing are typically characterized by biological exhaustion, cellular senescence, and an unreactive degenerative matrix rather than persistent acute inflammation, combining ESWT and PRP has emerged as a biologically plausible therapeutic concept [1,2,4-7]. It should be emphasized, however, that biological plausibility is not equivalent to demonstrated clinical synergy: at present, direct comparative clinical evidence remains limited to a small number of studies in selected pathologies. Recent clinical investigations have directly evaluated this combined approach in chronic patellar tendinopathy, lateral epicondylitis, partial-thickness rotator cuff tears, and aseptic nonunions [10-14].

This review covers all musculoskeletal conditions in which direct combination studies have been performed - chronic recalcitrant tendinopathies (patellar tendinopathy, lateral epicondylitis, and partial-thickness rotator cuff tears), biologically compromised bone healing (delayed union and nonunion), knee osteoarthritis, and carpal tunnel syndrome - and notes explicitly that for other prevalent conditions such as Achilles tendinopathy and plantar fasciitis, direct combination studies remain absent. Acute or postoperative tissue injuries are noted as an emerging but currently unreviewed frontier.

The objective of this selective narrative review is to synthesize the theoretical mechanisms, the direct and indirect clinical evidence, administration sequencing, and practical recommendations for ESWT and PRP combination therapy in these musculoskeletal conditions.

## Review

Search strategy and methodology

This article is a selective narrative review. A literature search of the PubMed/MEDLINE database was performed covering peer-reviewed articles published from inception through September 2026, and the search was rerun during revision to capture newly published studies. The search strategy utilized combinations of Medical Subject Headings (MeSH) terms and free-text keywords connected by Boolean operators (AND, OR): (“extracorporeal shock wave therapy” OR “ESWT” OR “shockwave therapy” OR “focused shock wave” OR “radial shock wave”) AND (“platelet-rich plasma” OR “PRP” OR “platelet-rich fibrin” OR “autologous conditioned plasma”) AND (“musculoskeletal” OR “tendinopathy” OR “patellar tendinopathy” OR “lateral epicondylitis” OR “rotator cuff” OR “Achilles tendinopathy” OR “plantar fasciitis” OR “nonunion” OR “delayed union” OR “pseudoarthrosis” OR “osteoarthritis”).

The inclusion criteria encompassed: (i) peer-reviewed original clinical trials (randomized controlled trials (RCTs), non-randomized prospective comparative studies, retrospective cohort studies, and representative case reports) evaluating the combination of ESWT and PRP or directly comparing both modalities in musculoskeletal disorders; (ii) systematic reviews and meta-analyses addressing the biological mechanisms or clinical outcomes of ESWT and PRP, used as supporting context; and (iii) articles published in the English language or with full peer-reviewed English translations available. The exclusion criteria comprised: (i) non-musculoskeletal applications (e.g., urological lithotripsy, erectile dysfunction, cardiac ischemic conditioning, dermatological wound healing); (ii) studies lacking detailed clinical outcome data; (iii) non-peer-reviewed conference abstracts, commentaries, or preprints; and (iv) articles with unverifiable data sources.

This review is a selective narrative synthesis rather than a systematic review. Publications were purposively selected by the author to represent the range of direct and indirect evidence available for each condition. No duplicate screening, study-selection flow diagram, or formal risk-of-bias assessment was applied, and the selection should not be assumed exhaustive. The clinical evidence synthesis is based on original primary studies (randomized trials, comparative cohorts, and case reports); systematic reviews and meta-analyses are used as supporting context only. Mechanistic and clinical background may draw on established review literature; such reviews were not part of the clinical evidence synthesis and did not contribute to the conclusions. Because the literature search and study selection were performed by a single author, selection bias is a potential limitation of this review.

Mechanistic overview of ESWT

ESWT involves the generation of biphasic acoustic pressure waves characterized by a rapid positive pressure rise (focal pressures of 50-80 MPa within <10 ns), followed by a slower negative pressure phase; the resulting acoustic cavitation is considered a major contributor to the biological effects [3]. Clinical devices utilize electrohydraulic, electromagnetic, or piezoelectric energy sources to generate fESWT, or pneumatic mechanisms to generate radial shock waves (rESWT, dispersion pressure waves) [1,3]. fESWT delivers maximal acoustic energy at a target deep within the tissues, whereas radial pressure waves are lower-intensity waves whose maximum energy occurs at the skin applicator surface [3]. Through mechanotransduction, mechanical stimulation of cells triggers downstream biological responses, including protein biosynthesis, cellular proliferation, and angiogenic factor expression [1-3]. As detailed in the review literature, ESWT upregulates pro-angiogenic mediators (e.g., VEGF, endothelial nitric oxide synthase (eNOS), proliferating cell nuclear antigen (PCNA)) and promotes mesenchymal stem cell recruitment and osteogenic differentiation [1,2]. Unlike urological shock wave lithotripsy designed for stone disintegration, musculoskeletal ESWT utilizes low- to medium-energy flux densities to stimulate regenerative and reparative pathways [1]. Throughout this review, focused and radial devices are distinguished wherever the source studies report them.

Mechanistic overview of PRP

PRP functions as an autologous bioactive scaffold and reservoir of concentrated growth factors and cytokines [4,5]. Sheean et al. underscored that clinical reporting of PRP must explicitly document baseline and final platelet concentrations, activation state, leukocyte composition, neutrophil proportions, and red blood cell counts [4]. Leukocyte-P-PRP is generally favored in intra-articular and delicate tendon applications to mitigate acute pro-inflammatory metalloproteinase release, whereas L-PRP may stimulate robust antimicrobial and chemotactic signaling in selected chronic degenerative or infected settings [5,6]. The multi-stage release of growth factors (PDGF-AA/BB/AB, TGF-β1/2, VEGF, bFGF, IGF-1) promotes local stem cell recruitment, tenocyte and chondrocyte proliferation, extracellular matrix type I collagen deposition, and neovascularization [4,6]. Clinical systematic reviews demonstrate that the efficacy of PRP monotherapy is highly pathology-specific: it demonstrates consistent therapeutic value in chronic patellar tendinopathy and lateral epicondylitis, whereas efficacy in Achilles tendinopathy and rotator cuff disease remains heterogeneous due to variable tissue biology and preparation methods [7-9].

Biological rationale for ESWT and PRP combination

The biological rationale for combining ESWT and PRP is rooted in their hypothesized complementary actions across distinct phases of tissue repair (Figure 1) [1,2,4-7]. This framework represents biological plausibility; whether the proposed interaction translates into clinically meaningful synergy can only be answered by direct comparative studies, which are reviewed below.

**Figure 1:**
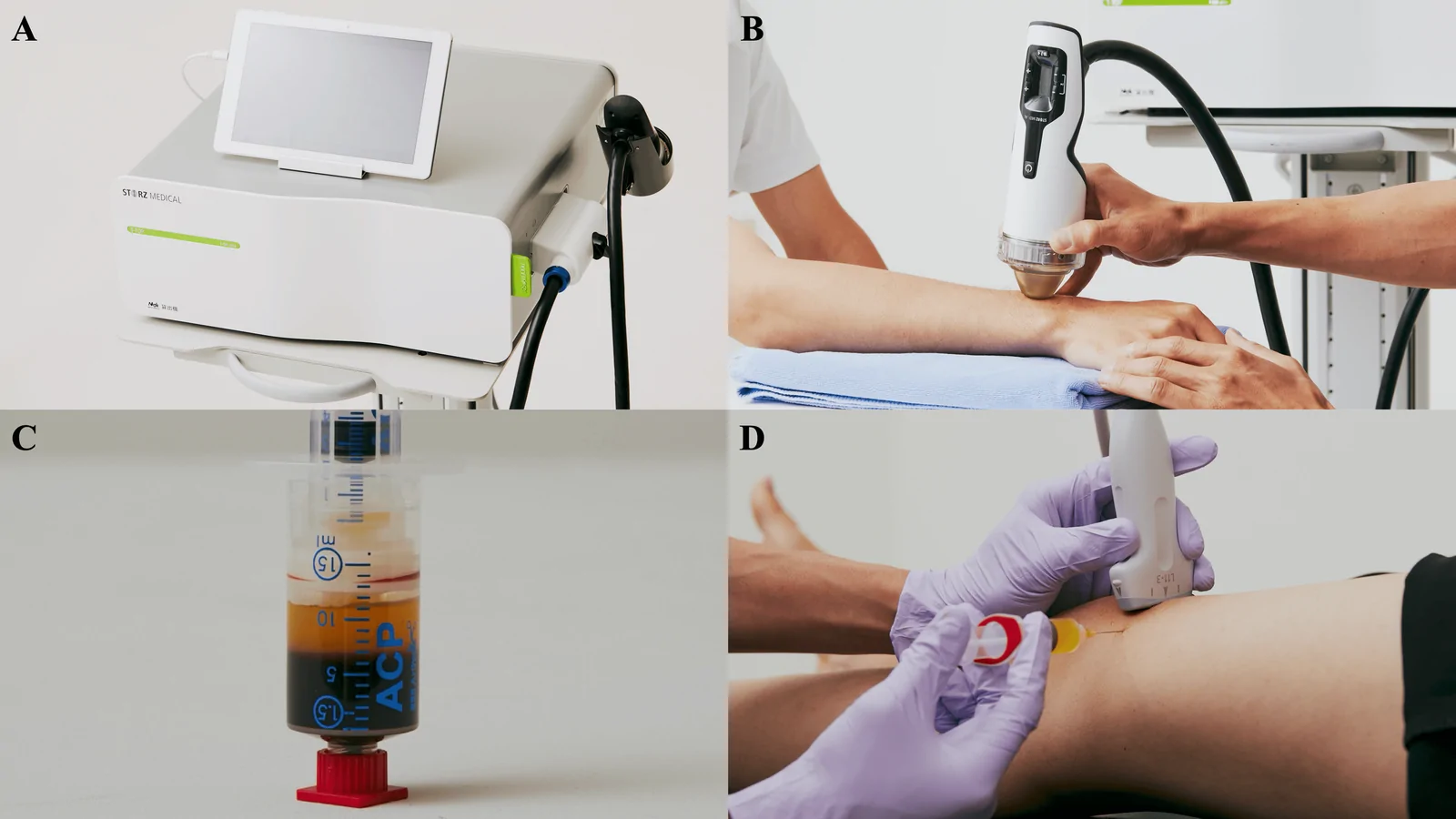
Extracorporeal shock wave therapy (ESWT) and platelet-rich plasma (PRP) A, B: An ESWT device and its clinical application for delivering acoustic shock waves to the affected musculoskeletal tissue. C, D: Preparation of autologous PRP by centrifugation and subsequent injection into the target lesion. Note: This photograph was taken during the authors’ clinical scene and depicts a real clinical setting.

In chronic tendinopathies and nonunions, tissue healing is arrested in a low-reactivity state. ESWT acts as a physical ‘priming’ stimulus shown to promote local tissue reactivity - including mesenchymal stem cell recruitment, upregulation of angiogenic growth factors, and neovascularization [1] - and subsequent or concurrent administration of PRP delivers concentrated autologous growth factors that may act on this primed environment, supporting cellular proliferation and extracellular matrix organization [4-7]. However, the major clinical barrier to universal adoption is the absence of standardized protocols regarding energy parameters, PRP preparation methods, and treatment intervals [2,4-7,10-14].

Clinical evidence in chronic tendinopathy

Patellar Tendinopathy

Patellar tendinopathy (‘jumper’s knee’) is among the most thoroughly investigated conditions for both monotherapies [8,15,16] and combination regimens [10,11]. Vetrano et al. conducted a seminal RCT in 46 athletes comparing two ultrasound-guided autologous PRP injections (spaced two weeks apart; n = 23) with three sessions of fESWT (2,400 impulses per session, EFD 0.17-0.25 mJ/mm²; n = 23) [15]. Both cohorts achieved significant clinical improvements at 2, 6, and 12 months; however, the PRP group achieved statistically superior Victorian Institute of Sport Assessment-Patella (VISA-P) and visual analog scale (VAS) scores at 6 and 12 months, indicating durable long-term symptom control with PRP monotherapy in that trial [15]. In a systematic review and meta-analysis of 70 studies involving 2,530 patients, Andriolo et al. reported that eccentric exercise provided the best short-term results, while multiple PRP injections offered more satisfactory long-term outcomes [16]. These monotherapy and meta-analysis data provide indirect context for the combination studies summarized below.

Direct clinical evidence for combining both modalities was provided by Jhan et al. in an RCT of 33 athletes with chronic refractory patellar tendinopathy randomized to PRP monotherapy (single 5 mL autologous PRP injection, prepared with an Arthrex ACP Double-Syringe System from 15 mL of whole blood; platelet concentrations of 300,000-1,200,000 platelets/μL with reduced leukocyte content; administered under ultrasound guidance using a 21G needle) versus combination therapy (the same PRP injection followed one week later by a single session of fESWT delivered with a DUOLITH SD1 device (Storz Medical): 1,350 impulses, EFD 0.2 mJ/mm², 4 Hz) [10]. Over a 12-month follow-up, both groups exhibited significant functional and pain improvements. Notably, the combination cohort experienced faster pain alleviation at one month only, with intergroup differences narrowing by 12 months; the benefit was thus limited to early pain relief and was not sustained across all outcome domains or time points [10]. For competitive athletes, rapid symptom resolution and accelerated return-to-play represent critical clinical endpoints, and patellar tendinopathy is therefore among the most evidence-supported indications for combination therapy [10,11].

Shimizu reported a three-arm exploratory randomized comparative study (pilot trial) in 10 competitive athletes with chronic patellar tendinopathy allocated to PRP monotherapy (n = 5; three leukocyte-poor PRP injections, mean 5.18 mL, ultrasound-guided, at two-week intervals), rESWT monotherapy (n = 2; diffuse-type device: 2,500 shocks, 2.4-4.0 bar, 8 Hz, once weekly for three sessions), or combined therapy (n = 3; both protocols administered concurrently over the same period) [11]. All participants followed a standardized progressive eccentric rehabilitation program. Over six months, all clinical outcomes (VAS, VISA-P, IKDC, Lysholm, and KOOS) improved significantly over time in every group without significant between-group differences in patient-reported scores. However, quantitative ultrasonography demonstrated significantly greater reductions in hypoechoic lesion area (p = 0.031) and intratendinous lesion length (p = 0.015) at six months favoring the combination group, with moderate-to-large effect sizes (ε² = 0.34-0.37), and MRI axial lesion thickness showed a similar pattern (p = 0.046) [11]. Given the exploratory pilot design and very small arm sizes (n = 2 and n = 3), these findings should be interpreted as hypothesis-generating structural signals requiring confirmation in adequately powered trials.

Lateral Epicondylitis

Chronic lateral epicondylitis (‘tennis elbow’) involves microtrauma and angiofibroblastic tendinosis of the common extensor origin [8,12,17]. Şahbaz et al. conducted a prospective randomized controlled study in 74 patients comparing exercise alone (n = 24), ESWT (one session weekly for three weeks) plus exercise (n = 25), and PRP (single injection) plus exercise (n = 25), all with a three-month home stretching and eccentric program [17]. At six-month follow-up, the PRP plus exercise group reported lower activity-related pain than the exercise-only group, and the authors concluded that PRP combined with exercise appeared superior to exercise or ESWT in terms of pain and functionality; no between-group superiority of ESWT over exercise alone was reported in the study abstract [17].

To rigorously assess additive combination efficacy, Akçin et al. conducted a prospective, randomized sham-controlled ultrasonographic trial in 91 patients allocated to: (i) PRP + ESWT + exercise, (ii) sham-PRP + ESWT + exercise, or (iii) ESWT + exercise alone [12]. The injection solutions were prepared by an unblinded researcher, whereas the patients, the administering physician, and the outcome evaluator were blinded to group allocation. A single ultrasound-guided PRP injection (approximately 3 mL; T-LAB kit, providing a 4.75-fold platelet concentration) was administered in combination with three weekly ESWT sessions (2 bar, 8 Hz, 2,000 pulses per session), applied in circular movements over the most tender area over the epicondylar region with the Swiss DolorClast Master device (Electro Medical Systems), alongside a three-month daily home program of stretching and eccentric strengthening exercises [12]. At 4 and 12 weeks, the combination cohort showed superior activity and night pain on the VAS compared with both comparator groups, with no significant difference in pain at rest. QuickDASH scores at 12 weeks were superior to both groups; PRTEE function was superior in the short term, and all PRTEE parameters at 12 weeks were superior to the ESWT-only group. Handgrip strength was superior to both groups at 12 weeks, whereas isokinetic wrist flexion/extension peak torque was superior only to the sham-PRP group [12]. Ultrasonographic common extensor tendon thickness and total ultrasound scale scores improved in all groups without significant between-group differences, indicating that the short-to-medium-term advantage of combination therapy was confined to symptomatic relief and functional muscle performance rather than tendon structure [12].

Rotator Cuff Tendinopathy and Partial-Thickness Tears

In rotator cuff disorders, clinical efficacy varies considerably across specific pathological subtypes (calcific tendinitis, non-calcific tendinosis, partial tears, and post-repair augmentation) [3,7,9]. While ESWT monotherapy demonstrates high efficacy in calcific rotator cuff tendinopathy [1,3], the meta-analysis by Hamid and Sazlina of eight RCTs showed that PRP injection was safe and effective for long-term pain control and shoulder function, although the included studies varied considerably in PRP preparation and comparators [9].

Kuo et al. evaluated the combination of ESWT and PRP in a prospective randomized comparative study of 55 patients with symptomatic partial-thickness rotator cuff tears randomized to autologous PRP monotherapy (n = 27; RegenKit THT system (RegenLab); 10 mL of venous blood; subacromial injection via a posterolateral landmark-based approach without ultrasound guidance, as explicitly stated by the authors) or PRP injection followed one week later by a single one-hour session of ESWT delivered with the Orthospec device (Medispec; 3,000 impulses, 24 kV, energy flux density 0.32 mJ/mm²) [13]. While both cohorts showed significant gains in Constant-Murley scores, VAS pain, and active range of motion (ROM) over six months, the combination group demonstrated significantly superior active forward flexion and abduction at one month, as well as greater total active ROM at six months [13]. Serum proteomic profiling revealed significant downregulation of pro-inflammatory alarmins S100A8 and S100A9 in the combination group at one month, suggesting an active anti-inflammatory biological modulation [13]. As in the other tendinopathy trials, the functional advantage emerged at selected time points (one-month forward flexion/abduction and six-month total ROM) rather than uniformly across all outcomes [13].

Clinical evidence in biologically compromised bone healing

Aseptic Nonunion and Delayed Fracture Healing

Impaired bone healing represents a substantial clinical challenge. In a systematic review of ESWT for delayed union and nonunion fractures across 30 studies, Willems et al. reported overall bone union rates of 86% for delayed unions, 73% for nonunions, and 81% for post-surgical nonunions [18]. Longo et al. reviewed tissue-engineering strategies for pseudoarthrosis, concluding that while surgical revision with autologous bone grafting remains the standard of care, biophysical adjuncts including ESWT and PRP offer promising non-operative alternatives [19].

The strongest direct clinical evidence for combined ESWT and PRP in bone pathology was established by Cen et al. in a comparative study of 60 patients with aseptic nonunion of long bones (tibia, femur, humerus, radius, ulna; 55 completed follow-up) [14]. PRP was prepared with a dedicated kit from approximately 45 mL of sodium citrate-anticoagulated venous blood by two centrifugation steps at 3,400 rpm to a final volume of 8-10 mL (mean platelet concentration approximately 420% of baseline, or 780,000 platelets/mL) and was activated with 10% calcium chloride and bovine thrombin (300 IU); under B-ultrasound guidance, fibrous scar tissue at the fracture gap was loosened with a needle and PRP was injected into the nonunion area once weekly for three weeks. rESWT (device class identified by the review author; the source reports bar-based settings only and does not name the device class or an energy flux density) was aimed at the fracture end, avoiding internal fixation, vessels, and nerves (shock pressure 2-4 bar; frequency 10 Hz; eight minutes per reference point at two reference points over the nonunion area; once weekly for three sessions, with the first ESWT session administered two days after the PRP injection), after application of a topical analgesic emulsion (Futalin) to the skin. The combination group achieved a statistically superior overall bone union rate (92.59% (25/27) vs. 71.43% (20/28), p < 0.05; an absolute difference of approximately 21 percentage points) and a shorter mean union duration (16.3 ± 5.2 weeks vs. 21.5 ± 3.7 weeks, p < 0.05) [14]. Radiographic callus formation (Lane-Sandhu) scores were significantly higher in the combination cohort at 8, 12, 16, 20, and 24 weeks, with no significant between-group difference at four weeks; Johner-Wruhs functional outcomes were significantly superior [14]. However, effect sizes and confidence intervals were not consistently available from this retrospective comparative study, and the results should be interpreted as suggestive of an additive effect rather than definitive evidence of synergy.

Stress Fracture Nonunion and Small Bone Defects

Omodani reported a successful application of combined ESWT and PRP in a 15-year-old competitive female sprinter suffering from a painful nonunion of a proximal phalanx stress fracture of the great toe, symptomatic approximately three months after initial conservative management [20]. Following a combined protocol in which PRP (Arthrex ACP Double Syringe System, 15 mL of blood) was injected under ultrasound guidance (2 mL subperiosteally at the fracture site and 2 mL into the metatarsophalangeal joint) and fESWT (DUOLITH SD 1, Storz Medical; 2,500 pulses, 0.25 mJ/mm², delivered every two weeks for a total of four sessions), complete radiographic bony consolidation was achieved six weeks after the start of treatment, and the athlete returned to competition at seven weeks [20]. In a systematic review of platelet gel in bone reconstruction, Lin reviewed platelet gel for bone defect reconstruction and nonunion, reporting that most studies combined platelet gel with bone grafts with positive results, and that the single study applying platelet gel combined with ESWT likewise reported positive findings [21]. However, direct clinical evidence in large structural bone defects and post-osteotomy nonunions remains in an exploratory stage [19,21].

Clinical evidence in knee osteoarthritis

Knee osteoarthritis is among the conditions for which a direct three-arm combination study has been performed. Su et al. investigated combination therapy in a randomized study of 180 patients with symptomatic knee osteoarthritis (Kellgren-Lawrence grades I-II, balanced between groups at baseline) randomized in equal groups of 60 to: (i) intra-articular autologous PRP alone (4 mL per injection, prepared from 40 mL of venous blood by density-gradient centrifugation; platelet concentration not reported; injected via a parapatellar approach under local anesthesia), (ii) ESWT alone, or (iii) intra-articular PRP injection followed 10 minutes later by ESWT, with all interventions administered once weekly for five sessions. ESWT was delivered with a MASTERPULS MP-100 device (STORZ Medical) - a radial pressure wave device per the manufacturer’s product line - targeted to osteophytic sites identified on radiographs and at tender points while avoiding major vessels and nerves, at an energy flux density of 0.12-0.20 mJ/mm² with 1,000-2,000 impulses per session; the application frequency was not reported [22]. Evaluations were performed before treatment and at one, three, and five weeks after the first treatment, using the VAS, Lequesne index, WOMAC, and knee joint ROM [22]. All groups improved significantly over time, and the combination group was reported to be superior to both monotherapy groups in VAS, Lequesne, and WOMAC scores [22]. This trial provides direct - although short-term and single-study - support for combined ESWT+PRP in degenerative joint disease, and warrants confirmation in larger trials with longer follow-up.

Clinical evidence in moderate carpal tunnel syndrome

Moderate carpal tunnel syndrome has been studied in a double-blind, placebo-controlled trial of combined ESWT+PRP. Chang et al. enrolled 40 patients (4 men, 36 women), all of whom completed the study; all received a single ultrasound-guided PRP injection at baseline, and two weeks later the intervention group (32 wrists) received one session of rESWT while the control group (32 wrists) received sham ESWT [23]. Evaluations were performed at baseline and at one, three, and six months after the PRP injection, with the Boston Carpal Tunnel Questionnaire (BCTQ) as the primary outcome and electrophysiological studies and median nerve cross-sectional area as secondary outcomes [23]. The combination group showed significantly better BCTQ symptom scores at one month (mean change ± SE, -11.47 ± 1.18 vs. -7.06 ± 1.26; p = 0.013) and distal motor latency at three months (-0.59 ± 0.09 vs. -0.30 ± 0.09; p = 0.031), but was not superior to PRP alone at any other time point, and the authors concluded that combined PRP and one-session rESWT was not superior to PRP alone in moderate carpal tunnel syndrome [23]. This neutral result is an essential element of the evidence base: it demonstrates that combination therapy does not confer universal additive advantages across all musculoskeletal and nerve conditions, and it cautions against extrapolating the positive signals in tendinopathy, bone healing, and osteoarthritis to other tissue types without direct trial data [23].

Administration sequencing and treatment protocols

A standardized consensus regarding the timing, sequencing, and dosing parameters of combined ESWT and PRP has not yet been established in clinical practice [2,4-7,10-14,20,22,23]. Published clinical trials reflect two distinct structural approaches: (i) sequential administration and (ii) concurrent multi-session packages. In the patellar tendinopathy trials, Jhan et al. delivered a single PRP injection followed one week later by a single fESWT session [10], whereas Shimizu et al. administered rESWT and PRP injections concurrently over the same three-week period [11]. In lateral epicondylitis, Akçin et al. combined a single ultrasound-guided PRP injection with three weekly ESWT sessions (Swiss DolorClast Master; 2 bar, 8 Hz, 2,000 pulses) and a three-month home eccentric exercise program [12]. In partial rotator cuff tears, Kuo et al. administered a single ESWT session with the Orthospec device (Medispec) one week after PRP [13], and Cen et al. injected PRP once weekly for three weeks and applied ESWT (2-4 bar, 10 Hz, eight minutes per reference point) weekly for three sessions beginning two days after the first PRP injection for long-bone nonunions [14]. Conversely, Su et al. injected intra-articular PRP followed 10 minutes later by rESWT (MASTERPULS MP-100; EFD 0.12-0.20 mJ/mm², 1,000-2,000 impulses per session), with both administered once weekly for five sessions for knee osteoarthritis [22]. In the index case of great-toe stress-fracture nonunion, fESWT (2,500 pulses, 0.25 mJ/mm²) was applied every two weeks for four sessions, with ultrasound-guided PRP injection (2 mL at the fracture site and 2 mL intra-articularly) [20].

From a biological perspective, delivering PRP first allows growth factors to act on the target tissue, which may subsequently be potentiated by acoustic shock wave mechanotransduction [1,2,4-7]. Alternatively, applying ESWT prior to PRP has been hypothesized to ‘prime’ the target tissue and optimize PRP uptake [1,2]. While the ‘PRP-first, followed by ESWT within one to two weeks’ strategy represents the most frequently tested clinical paradigm, future comparative head-to-head trials are required to determine true sequence superiority.

Summary of published evidence and clinical synthesis

Table 1 summarizes the key clinical studies investigating combined ESWT and PRP therapy, including the specific ESWT protocols used in each study.

**Table 1:** Major clinical studies on combination therapy (with ESWT protocols) ESWT and PRP protocol parameters for the Cen, Shimizu, Akçin, Kuo, Omodani, and Su studies were extracted verbatim from the full texts (the four primary studies provided during revision, plus the open-access full texts); the Chang et al. parameters were extracted from the published abstract. “Not reported” indicates that the parameter was not documented in the source study; no parameter was inferred or transferred from other studies. Manufacturer classifications: the Orthospec (Medispec) is an electrohydraulic system; the MASTERPULS MP-100 (STORZ Medical) and the Swiss DolorClast Master (Electro Medical Systems) belong to the manufacturers’ radial shock wave product lines. The Akçin and Su source studies do not themselves use the terms “focused” or “radial”; the Akçin study reports bar-based settings only, without an energy flux density in mJ/mm². The radial classification of the device in the Cen et al. study was provided by the review author; the Cen source text does not use the terms “focused” or “radial” - it reports bar-based dosing without naming the device class or manufacturer and states only that the machine was aimed at the fracture end. NR: Not Reported; EFD: Energy Flux Density; ACP: Autologous Conditioned Plasma; BCTQ: Boston Carpal Tunnel Questionnaire; EFD: Energy Flux Density; ESWT: Extracorporeal Shock Wave Therapy; fESWT: Focused Extracorporeal Shock Wave Therapy; LP-PRP: Leukocyte-Poor Platelet-Rich Plasma; PRP: Platelet-Rich Plasma; rESWT: Radial Extracorporeal Shock Wave Therapy; ROM: Range of Motion; US: Ultrasound; VAS: Visual Analog Scale; WOMAC: Western Ontario and McMaster Universities Osteoarthritis Index

Pathology	Study	Design	Subjects	ESWT modality and protocol	PRP preparation and injection	Main Results	Interpretation
Patellar tendinopathy	Jhan et al. [10]	RCT	33 athletes; PRP alone vs. PRP + ESWT	Focused (DUOLITH SD1, Storz Medical); 1,350 impulses; EFD 0.2 mJ/mm²; 4 Hz; single session, 1 week after PRP; no local or regional anesthesia; skin contact via surgical lubricant	Arthrex ACP Double-Syringe System; 15 mL whole blood; 300,000-1,200,000 platelets/μL (2-3x baseline), leukocyte-reduced; no local anesthetic before injection; single 5 mL injection, ultrasound-guided (21G)	Both groups improved; pain reduction at 1 month was faster in the combination group	Suggests an additive early response
Patellar tendinopathy	Shimizu [11]	Three-arm randomized comparative study (pilot)	10 athletes; PRP alone (n=5) vs. radial-type ESWT alone (n=2) vs. concurrent combination (n=3)	Radial-type (diffuse-type; Physio Shock Master, Sakai Medical); 2,500 shocks; 2.4-4.0 bar; 8 Hz; once weekly x3	Arthrex ACP double-syringe system (Arthrex, Naples, FL); ~15 mL blood; 5-min centrifugation; LP-PRP; platelet count and activation not reported; mean 5.18 ± 0.8 mL; three ultrasound-guided injections (22G) at 2-week intervals, concurrent with the ESWT course	All clinical scores improved over time without between-group differences; US hypoechoic lesion area and lesion length at 6 months superior in the combination group (p=0.031/0.015, ε²=0.34-0.37)	Hypothesis-generating structural signal from a small pilot
Lateral epicondylitis	Akçin et al. [12]	Prospective randomized sham-controlled trial	91 cases (n=30/29/32); PRP+ESWT+exercise vs. sham PRP+ESWT+exercise vs. ESWT+exercise	Radial-type (Swiss DolorClast Master, EMS); 2 bar; 8 Hz; 2,000 impulses per session; once weekly x3; circular movements over the most tender area	T-LAB kit (T-Biotechnology); 10 mL blood; 1,500 x g for 8 min; 4.75-fold platelet concentration; leukocyte status and activation not reported; single ~3 mL injection, ultrasound-guided (21G); sham group received 3 mL of 0.9% normal saline	Combination superior in activity/night VAS (vs. both), QuickDASH at 12 weeks (vs. both), PRTEE (12 weeks, vs. ESWT-only), and handgrip strength (12 weeks, vs. both); isokinetic torque vs. sham-PRP only; no US difference	Symptomatic benefit appears relatively clear
Partial rotator cuff tear	Kuo et al. [13]	Prospective randomized comparative study	55 cases (combination 28 vs. PRP alone 27); PRP alone vs. PRP + ESWT	Electrohydraulic (Orthospec, Medispec); modality term not specified in source; 3,000 impulses; 24 kV (EFD 0.32 mJ/mm²); single hour-long session, 1 week after PRP; no local anesthetics; coupling via surgical lubricant	RegenKit THT (RegenLab); 10 mL blood; platelet concentration, injected volume, and activation not reported; single subacromial injection via a posterolateral landmark approach without ultrasound guidance and without local anesthesia (as stated by the authors)	Combination group showed better forward flexion/abduction at 1 month and better total ROM at 6 months	Suggests an additive effect at selected time points
Aseptic nonunion of long bones	Cen et al. [14]	Retrospective comparative study	60 cases; PRP alone vs. PRP + ESWT	Radial-type (per review-author identification; source does not name the device class or manufacturer and reports bar-based settings only); device aimed at the fracture end, avoiding internal fixation, vessels, and nerves; 2-4 bar; 10 Hz; 8 min per point x2 reference points; once weekly x3, starting 2 days after the first PRP injection; topical analgesic emulsion (Futalin) applied to the skin before ESWT	Shandong Weigao Xinsheng kit; ~45 mL blood with sodium citrate anticoagulant; two-step centrifugation (3,400 rpm for 15 min, then 10 min; chilled Kubota 9800); final volume 8-10 mL; ~420% of baseline (mean 780,000 platelets/mL); leukocyte status not reported; activated with 10% calcium chloride and bovine thrombin (300 IU, Fibriquik, BioMerieux); B-ultrasound-guided injection into the fracture gap after needle loosening of fibrous scar tissue (No. 9 needle); once weekly x3 weeks	Bone union rate 92.59% vs. 71.43%; shorter healing period	Strongest direct evidence in bone disorders
Great toe stress fracture nonunion	Omodani [20]	Case report	15-year-old female sprinter; ESWT + PRP	Focused (DUOLITH SD 1, Storz Medical); 2,500 pulses; 0.25 mJ/mm²; every two weeks, four sessions	Arthrex ACP Double Syringe System; 15 mL blood; 1,500 rpm for 5 min; final volume 4 mL; leukocyte-poor; platelet concentration not reported; ultrasound-guided (Aplio i700, Canon) 22G needle; 2 mL subperiosteally at the fracture site plus 2 mL into the metatarsophalangeal joint	Complete bone union and return to sport at 6 weeks	Hypothesis-generating but clinically suggestive
Knee osteoarthritis	Su et al. [22]	Randomized comparative study	180 cases (n=60 each); PRP alone vs. ESWT alone vs. combination	Radial-type (MASTERPULS MP-100, STORZ Medical) per manufacturer classification; source does not use the term “radial”; EFD 0.12-0.20 mJ/mm²; 1,000-2,000 impulses per session; frequency not reported; targeting by radiograph plus tender points at osteophytic sites, avoiding vessels and nerves; once weekly x5, 10 minutes after the PRP injection	Density-gradient (three-layer) centrifugation from 40 mL antecubital venous blood; 4 mL per injection; platelet concentration, leukocyte status, and activation not reported; intra-articular injection via the ‘knee-eye’ (parapatellar) approach under local anesthesia, needle advanced 2.0-2.5 cm with aspiration check; once weekly x5	Combination superior in VAS, Lequesne, and WOMAC at 1, 3, and 5 weeks	Direct three-arm evidence in degenerative joint disease; short-term benefit
Moderate carpal tunnel syndrome	Chang et al. [23]	Randomized double-blind placebo-controlled trial	40 patients (64 wrists; 32 per group); all received PRP; + rESWT x1 after 2 weeks vs. sham	Radial; device name, EFD, impulses, and frequency not reported; single session, 2 weeks after PRP	Preparation details not reported in the source; single ultrasound-guided injection at baseline	Superiority over PRP alone was not demonstrated, except for BCTQ at 1 month and distal motor latency at 3 months	Shows that combination therapy is not always advantageous

Beyond Table 1, the direct clinical evidence base for combined ESWT+PRP consists of only eight studies: two randomized studies in patellar tendinopathy [10,11], one sham-controlled RCT in lateral epicondylitis [12], one comparative trial in partial-thickness rotator cuff tears [13], one retrospective comparative study in aseptic long-bone nonunion [14], one case report in great-toe stress-fracture nonunion [20], one randomized study in knee osteoarthritis [22], and one double-blind RCT in moderate carpal tunnel syndrome [23]. Read together, these studies suggest possible additive benefits in early pain relief (patellar tendinopathy) [10], short-to-medium-term symptoms (lateral epicondylitis) [12], selected range-of-motion outcomes (partial rotator cuff tears) [13], short-term symptomatic relief in knee osteoarthritis [22], and union outcomes (nonunion) [14], set against a neutral result in carpal tunnel syndrome [23] and a hypothesis-generating ultrasonographic signal in the small patellar pilot [11]. All other works cited in this review - including monotherapy trials, monotherapy systematic reviews and meta-analyses, and mechanistic or background reviews - are used solely to provide context, to characterize evidence gaps, or to explain the biological rationale; they do not by themselves constitute evidence that combined ESWT+PRP is efficacious for any condition. Accordingly, no summary table beyond Table 1 is provided, to avoid implying a graded synthesis that the sparse direct evidence cannot support.

Discussion

The primary insight from this review is that combining ESWT and PRP constitutes a mechanobiological strategy [1,2,4-7] with a suggestive clinical signal in specific musculoskeletal pathologies [10-14]. Specifically, direct comparative studies suggest possible additive benefits in selected outcomes in chronic patellar tendinopathy, lateral epicondylitis, partial-thickness rotator cuff tears, aseptic long-bone nonunions, and knee osteoarthritis [10-14,22], although these studies vary in design, sample size, and risk of bias. These responsive conditions share a common pathophysiology: chronic tissue degeneration characterized by hypervascular or hypovascular dysrepair, extracellular matrix disorganization, and cellular unresponsiveness rather than acute unrestrained inflammation [1,2,4-7]. In this biological setting, ESWT-induced acoustic mechanotransduction promotes tissue reactivity and endogenous angiogenic signaling [1,3], which may create a more receptive environment for concentrated PRP growth factors to support matrix remodeling [4-7].

Analyzing specific clinical domains, Jhan et al. established that adding a single fESWT session to PRP accelerates one-month pain resolution in patellar tendinopathy [10], and Shimizu et al. reported a small pilot trial in which the combination was associated with superior six-month ultrasonographic structural outcomes [11]. In lateral epicondylitis, Akçin et al. demonstrated that triple therapy (PRP + ESWT + eccentric exercise) achieved superior activity and night pain relief compared with both comparator arms and superior functional scores in the short-to-medium term, although the isokinetic strength advantage was confined to comparison with the sham-PRP arm [12]. In the shoulder, Kuo et al. observed improved ROM at selected time points together with systemic downregulation of pro-inflammatory alarmins S100A8/A9 [13]. In bone pathology, Cen et al. documented a higher bone union rate (92.59% vs. 71.43%) and a 5.2-week reduction in mean healing time in long-bone nonunions; however, given the retrospective design and modest sample size, this finding supports an additive effect on union rate and time to union rather than definitive proof of biological synergy [14]. In knee osteoarthritis, Su et al. reported short-term symptomatic superiority of the combination over either monotherapy on VAS, Lequesne, and WOMAC scores [22].

Despite these encouraging findings, substantial evidence gaps and methodological barriers prevent ESWT+PRP from being recommended as a universal first-line standard of care. First, considerable heterogeneity exists in PRP preparation protocols, including baseline platelet enrichment ratios, leukocyte inclusion (P-PRP vs. L-PRP), activation techniques, and volume administered [4-7]. Second, ESWT parameters vary widely across studies regarding wave modality (focused vs. radial), energy flux density (low vs. medium vs. high energy), total pulse count (approximately 1,000-3,000 impulses per session, with one bone-healing study using time-based dosing of 8 minutes per treatment point), and focal depth targeting [1,2]. Third, for prevalent conditions such as chronic Achilles tendinopathy and plantar fasciitis, the literature remains dominated by trials comparing ESWT versus PRP - for Achilles tendinopathy, with insertional and midportion phenotypes typically distinguished and monotherapy comparisons suggesting comparable mid-term outcomes between fESWT and PRP injections - with an acute lack of direct combination studies [8,24-29]. Fourth, neutral results observed in carpal tunnel syndrome demonstrate that combination therapy is not universally effective across all tissue types [23]. Finally, concurrent co-interventions - most notably structured physical therapy and eccentric loading protocols - frequently confound the isolation of pure biophysical synergy [12].

The clinical position of ESWT+PRP should also be understood relative to competing and complementary regenerative and minimally invasive options. Dextrose prolotherapy has a long history in chronic musculoskeletal pain [30], high-volume image-guided injection has been evaluated in recalcitrant patellar tendinopathy [31], cell-based orthobiologic approaches have been proposed within tiered treatment algorithms [32], and ultrasound-guided nerve hydrodissection has been described for entrapment neuropathies [33]. These treatments target overlapping biological niches, although head-to-head comparisons with ESWT+PRP are lacking. The expanding use of ESWT beyond chronic disorders, including post-surgical and fracture-related settings, further suggests that the ‘priming’ model described here may not be the only biologically active context for combination therapy.

Based on current evidence synthesis, combination therapy should not be applied indiscriminately. Instead, clinicians may consider ESWT+PRP combination therapy in the following scenarios: (i) Chronic recalcitrant tendinopathy (patellar, lateral elbow, partial rotator cuff) failing at least three months of standard conservative care (eccentric exercise, activity modification, pharmacotherapy) [10-13]. (ii) High-demand athletes or occupational workers in whom accelerated symptom alleviation, rapid functional recovery, and early return-to-activity are paramount [10,12]. (iii) Aseptic, stable long-bone delayed unions or nonunions in which impaired biological reactivity is the primary etiology, after confirming mechanical stability [14,18]. (iv) Patients seeking to avoid invasive surgical intervention who understand the non-standardized nature of the protocol [14,19]. (v) Symptomatic knee osteoarthritis, as a short-term adjunctive option where injection-based regenerative therapy is considered, with the explicit caveat that the evidence derives from a single randomized study with short-term follow-up [22].

This review has several limitations. First, the literature search and study selection were performed by a single author, which constitutes a potential source of selection bias. Second, as a selective narrative synthesis, the review does not include a systematic search across multiple databases, duplicate screening, or formal risk-of-bias assessment, and publication bias cannot be excluded. Third, the small number and heterogeneity of direct combination studies preclude any quantitative synthesis.

## Conclusions

Combining ESWT and PRP is a biologically plausible strategy for chronic recalcitrant tendinopathy and biologically compromised bone healing. Direct comparative studies suggest possible additive benefits - faster early pain relief in patellar tendinopathy, better short-to-medium-term symptomatic outcomes in lateral epicondylitis, selected range-of-motion gains after partial rotator cuff tears, higher union rates in aseptic long-bone nonunions, and short-term symptomatic benefit in knee osteoarthritis - although these benefits are limited to selected outcomes and time points rather than consistent superiority, and a double-blind trial in moderate carpal tunnel syndrome was neutral. Direct evidence remains limited or absent in Achilles tendinopathy, plantar fasciitis, and large bone defects. The combination should therefore be regarded as a promising, specialized second-line option for refractory conditions rather than a universal standard of care, pending standardized PRP characterization, modality-specific and fully reported ESWT dosing parameters, and adequately powered multicenter trials.
